# The Hospital. Nursing Section

**Published:** 1903-03-28

**Authors:** 


					The Hospital.
Wuwtng Section. -L
Contributions for this Section of "The Hospital" should be addressed to the Editor, "The Hospitab"
Nursing Section, 28 & 29 Southampton Street, Strand, London, W.O.
NO. 861.?Vol. XXXIII. SATURDAY, MARCH 28, 1903.
"Motes on IRcwfi from tbe IRursing Morl&.
THE ENGLISH PIANIST.
Miss Grace Smith, the young pianist who had
the honour of playing before the Queen at uc ing
ham Palace on Monday afternoon, is the daughter ot
a clergyman of the Church of England, and was
educated at St. Margaret's School, Bushey. -tier
special talent was first noticed when she was only
nine years old, and since the age of twelve she as
been under careful training, first by Mile. Colmache,
next by Signor Busoni at Weimar, and by Mme.
Carreno at Berlin. Miss Smith's training has just
been completed. We would suggest that those
interested in concerts and entertainments for charity
should at once try and secure Miss Grace Smith, who
is likely to have many engagements this season,
*ow Queen Alexandra has taken her under her
protection. Her Majesty was delighted with JYiiss
Grace Smith's playing, recognising at once, w?n
her great knowledge of the subject, that Miss Smith s
execution is really very remarkable. We un er
the Queen is convinced Miss Grace Smi
great future before her. In these days everybody
"^ill welcome the advent of an English pianis o
first rank, and desire to hear and support a Jady wno
^ one of our own nation. Her address is -
Terrace, W.
THE PRINCESS OF WALES AND THE BRITISH
LYING-IN HOSPITAL.
The Princess of Wales has consented to J
?pen the new Nurses' Home at the Bri 18 ,
Hospital in Endell Street, on June 8th. ?
IS approaching completion, and should b nuDiis
occupation early in May, when the session P P
begins. It will accommodate twenty nurses,
large dome-roofed dining-room lighted from the ceil-
ing, and artistically designed with corner cupboards,
and a pretty green-tiled fire-place. There ar&
separate sitting-rooms for the nurses and pupils, and
the bedrooms, which are very light considering that
there ia not much open space on either side of the-
building, are being colour-washed, a different colour
for each of the three floors. The heating is carried
out by means of radiators in the passages. The com-
pletion of the Home will enable two additional wards>
to be thrown open for patients. The matron's sitting,
and bedrooms will remain in the hospital, which is.
connected by a passage with the new home.
PRINCESS HENRY OF BATTENBERG AND
DISTRICT NURSING.
Tiie annual meeting of the Hyde District Nursing:
Association took place last week, Princess Henry of
Battenberg, Governor of the Isle of Wight, in the-
chair. The report, which was read, recorded that an,
addition had been made to the home, and that the
extra accommodation had materially improved the?
comfort and well-being of the nurses, who paid
nearly 8,000 visits during the year, an increase of
669 on the previous twelve months. In the course?
of the year the visiting inspector from Queen Vic-
toria's Jubilee Institute made a very satisfactory
report as to the management of the home. The-
financial statement having been adopted, and
the executive re-elected, a cordial vote of thanks
to Princess Henry for her presence and for th&
practical interest taken by her in the poor, was
passed. After the meeting Her Royal Highness
visited the homeland the district nurses had the*
gratification of being presented to her.
March 28, 1903. THE HOSPITAL. Nursing Section. 353
DEATH OF MISS L. M. GORDON.
We much regret to hear of the death of Miss
L. M. Gordon, who only in October last resigned
the post of matron of St. Thomas's hospital. The
sad event took place on Sunday, at 29 Pembroke
Crescent, Hove, Brighton. It was hoped by all the
numerous friends of Miss Gordon that, though her
retirement was due to failing health, she would be
able to enjoy her well-earned rest for some time to
come, and the announcement of her decease will come
as a shock to many. A full account of her active
career and successful work appeared in The Hospital
of July 26, 1902. We are sure that profound sym-
pathy will be felt by nurses generally, and by St.
Thomas's nurses in particular, with the sisters of
Miss Gordon in the bereavement which they have
sustained.
A NURSES' HOME BADLY NEEDED.
An appeal is being circulated on behalf of the
nurses of the Royal Hospital for Diseases of the
Chest, City Road, London, in which it is
pointed out that in 1899 the committee of King
Edward's Hospital Fund and the medical staff of
the hospital condemned the accommodation for the
nursing staff, and urged the provision, with as little
delay as possible, of a suitable home for the nurses,
separated from the main hospital building. Towards
this object the committee of the King's Fund last
year contributed a sum of ?500, and a convenient
freehold site adjoining the hospital has been pur-
chased at a cost of ?2,000. As soon as the necessary
funds are forthcoming this work will at once b^
proceeded with. The sisters and nurses are at
present sleeping immediately over the wards, ob-
viously a very bad arrangement; and not only the
Lord Mayor, who presided, but almost all the
speakers at the annual court last week insisted on
the necessity for altering the state of affairs. Plans
have been prepared by Mr. Keith D. \oung, but
for lack of funds they have not yet been considered.
-A- sum of ?7,000 is required for the building and
furnishing of the home.
IS A NURSING HOME A NUISANCE?
The question whether a nursing home is a nuisance
has again been raised, this time in the Lancashire
Court of Chancery. An action was entered with the
view of preventing the continued existence of a
nursing home on the Park field Estate, a residential
suburb of Liverpool, which, it was alleged, was
calculated to be a nuisance and a source of annoy-
ance to the neighbourhood. It was also contended
that its establishment was a breach of certain
covenants. Witnesses were called to prove that
People in the neighbourhood objected to have invalids
going to and from the home, and also to be reminded
pf disease, which it was maintained, had a depress-
ing effect upon the residents. The possibility of con-
tagion was likewise suggested. This, however, was
disposed of by medical witnesses on the other side,
"^ho testi6ed that the home was of a high-class
character, and there was no likelihood of patients
suffering from infectious complaints being admitted
by mistake. It was mentioned that^ 15 patients
*re the maximum. Mr. Lawrence, K.C, averred
that the strength of the case for the defence was
that there was not even a fear of anything being
done that could properly be considered a nuisance.
In this view the Vice-Chancellor concurred, and
while admitting that a nuisance might grow up
within the meaning of the covenant, thought the
action to prove that it was one now, had failed on
every ground. It was, therefore, dismissed with
costs. We entirely endorse the judgment. A well-
conducted nursing home is not per se a nuisance any
more than a house containing a large family; it is
only when it ceases to be well conducted that it is
liable to become one.
THE "MARKET VALUE" OF A NURSE.
There was a curious discussion at the last meeting
of the Wimborne and Cranborne Guardians, when
the application of the master and matron for an
increase of salary was considered. The board, by a
majority of 12, rejected a resolution in favour of an
increase of ?o in the matrons salary, and an applica-
tion from the head nurse for an increase of ?5 a year
was brought before them. Several of the Guardians
supported this application, and the Chairman, who
said he regretted that the board had not given the
matron ?5 more, also advocated it. He affirmed
that " the market value of a nurse was more than
that of a master or a matron, and he hoped that the
Guardians would not run the risk of losing such a
valuable servant." The result was that only four
members voted against the proposal and 16 for it.
The Wimborne and Cranborne Board are therefore
clearly of the same opinion as their chairman, that
the " market value " of a nurse is more than that of
a master or a matron.
DEATH OF A NURSE FROM YELLOW FEVER.
A nursing sister who received the medal for
service in the South African campaign has, we
regret to learn, fallen a victim to yellow fever in
Brazil. Miss L. L. Watts was on the nursing staff
at the Strangers' Hospital, and contracted the disease
whilst attending a case at Nictheroy. She was ill
for only three or four days, and died at 11.30 p.m. on
the 25th of last month at the hospital. Some
British residents, anxious to pay a compliment to
her profession, had organised a little demonstration
in her honour, when death stepped in'and anticipated1
them. A large number of residents were present at
the funeral, the coffin being covered with wreaths
and natural flowers. Miss Watts was trained at
the Leeds General Infirmary, and was afterwards
night superintendent at the Nurses' Home, Frederick
Street, Belfast, and subsequently joined the Nurses'
Co-operation.
THE SUPERINTENDENT NURSE AND THE WORK-
HOUSE MASTER.
At a special meeting of the County of Surrey
Poor Law Officers' Association held at Croydon,
there was an animated discussion on the report of
the Departmental Committee on workhouse nursing.
The master of Epsom Workhouse said that if the
report was accepted, it would probably cause chaos
in the workhouse, "for if a superintendent nurse
had any complaint to make about the conduct of the
matron she was to take it to the master. He pitied
the poor master, for he would be placed literally
354 Nursing Section. THE HOSPITAL. March 28, 1903.
between the devil and the deep sea." Without
explaining whether he considered the superintendent
nurse the devil and the matron the deep sea, or vice
versd, Mr. White went on also to complain that, accord-
ing to the report, " a superintendent nurse could go to
the master and ask for anything she thought fit, no
matter how extravagant it might be, and the master
had to give it to her. That could not be tolerated
for one moment." But it will have to be tolerated if
the Local Government Board adopt the recommenda-
tion of the committee in reference to the authority
of a superintendent nurse. The superintendent
nurse at Croydon Infirmary urged that the dietary
of the nurses needed improvement ; and there is no
doubt with regard to diet that the superintendent
nurse should be able to ask for what she thinks fit,
even at the risk of exciting the consternation of the
master.
DORSET COUNTY HOME FOR NURSES.
The fact that the matron of the Dorset County
Home for Nurses had to refuse 44 private cases last
year shows that the institution is not only needed,
but is also appreciated. This would not, we think,
be so if a proposal at the annual meeting to employ
nurses with lower qualifications than the existing staff
possess had been entertained by the committee, which
includes several well-known medical men, who, to
quote the words of Captain Acland, were " dead
against it." Captain Acland also cited the failure
of a nurses' home in the same county which used to
.send out nurses of two classes ; and it is certain that
the Dorset County Home, which has won a reputa-
tion for supplying only first-class nurses, would
speedily lose the confidence it possesses if the retro-
grade suggestion were adopted. Another proposal
Tvas to reduce the fee of Is. a day which is charged
to the poor. The fee is not high for the services
Tendered, since the nurse is prepared to give her
whole time to the case. But it is more than the
poor can afford to pay, and we are disposed on
this point to agree with Canon Hankey that
as the institution was established mainly to benefit
the large middle-class, the efforts of the com-
mittee should be concentrated on this object.
The difficulty seems to be that extraneous financial
assistance is required, and that it can only be
obtained on the ground that the home undertakes
the nursing of the poor. But an organisation that
charges Is. a day for nursing cannot expect to be
regarded in the same light as a charity that sends out
a district nurse free of all cost.
UNFOUNDED CHARGE AGAINST A CORNISH
NURSE.
The Helston Board of Guardians very properly
inquired into a most serious charge against one of
the nurses in their employ. She was accused of
striking an inmate ; and it was, in fact, alleged that
she had given the woman two black eyes. The
visiting committee heard the evidence of the woman,
of her son who made the complaint, of two other
inmates, and of the nurse herself, with the result
that all but one member entirely exonerated the
nurse from blame in the matter. The dissenting
guardian said that his view was that the charge was
ct not proven." When, however, the report of the
committee came before the board they adopted it,
and the conclusion must therefore be that the
charge against the nurse, which, if sustained, would
have involved her instant dismissal, was quite
unfounded.
THE CONVERSION OF A CO-OPERATION INTO
A CHARITY.
With reference to our remark in a recent issue
respecting the position of the Glasgow and West of
Scotland Co operation of Trained Nurse3 that " the
power of the committee is now absolute," Mr. J. S.
Bannatyne writes us that "it would be an easy-
matter for the nurses, by application to the Court of
Session, to have the new rule reduced on the ground
that the executive have abused their position by
causing the nurses who had previously opposed the
rules to apologise for doing so and have thus intimi-
dated them from further opposition." Other grounds
on which the application might be made suggest
themselves to Mr. Bannatyne, but he believes that
the one given is quite sufficient to ensure reduction.
As to the expense, he admits that it would fall upon
the funds of the Co-operation. But he urges that,
as the nurses are not now to share in them, that
fact should not deter them from taking proceedings,
for his view is that proceedings would involve the
resignation of the executive, in which event "the
nurses would have the management of the funds
and property belonging to themselves in their own
hands." Another view, on the opposite side, is
given in our correspondence column by one of the
staff of the Co-operation.
SHORT ITEMS.
The s.s. Ortona, which arrived at Southampton
from Egypt last week, had on board Sister C. Ware,
of the Army Nursing Service, " on leave."?A sup-
porter of the Pontypridd Nursing Association, whose
name is not given, having promised ?1,000 towards
the provision of a nurses' home, the society is ap-
pealing for funds to furnish it.?A Nursing Associa-
tion has been formed in South Cramlington, Morpeth,
and a nurse engaged.?A mo3t successful entertain-
ment was given last week at the Cancer Hospital,
Fulham Road, to the in-patients, by Mrs. Read and;,
Miss Jordan, assisted by members of the Cripplegate
Club.?Princess Christian and Princess Victoria of
Schleswig-Holstein were present on Saturday at a
concert in Hampstead Town Hall in aid of the
Hampstead Nursing Association for tending the sick
poor in their own homes.?There will be a League of
Mercy meeting at Chelsea Town Hall on the evening
of Friday, April 3rd, Lord Cadogan in the chair.?-
An Association has been formed under the auspices
of Mrs. Hey wood Johnstone and others to train and
supply midwives for rural districts, either to add to
nursing associations already formed or to help very
poor parishes unable to support a nurse or midwife
without assistance.?The annual exhibition and sale
of Brabazon work has just taken place in the large
dining-room of the Norwich Workhouse under the
auspices of the Brabazon Committee, with Major
Crichton Browne as President. There was a large
and influential company, and the contents of the
stalls excited great interest.
March 28, 1903. THE HOSPITAL. Nursing Section. 355
ZEbe Hursing ?utloof?.
1 From magnanimity, all fear above;
From nobler recompense, above applause,
Which owes to man's short outlook all its charm."
GIFTS.
There are two points of view with regard to the
giving of gifts to nurses, and both points deserve
some consideration. For the question is undoubtedly
a debatable one, and one that comes up frequently in
?every nurse's life. To look at it with the eyes of the
public first: There is the man or woman who, having
received great kindness from a nurse, desires to mark
it with the gift of a gold watch or a leather bag, and,
should the rules of the institution forbid, they are
hurt, and probably give the present all the same.
The feelings are very tender and emotional when
?sickness and sorrow are in a household, and where
money is plentiful gratitude may like to express
itself in jewellery or books; but such feelings are very
evanescent, and it is possible that afterwards, if the
presents are accepted, the nurse may fall in the
estimation of the donor and be characterised as a
41 greedy thing." At the next case the nurse may go
"to money may be scarce and the strain of the cost of
the illness be already more than the friends can well
bear. What then are their thoughts probably when
the nurse shows with pride the beautiful gold watch
her last patient gave her 1 No wonder there is an
?occasional remonstrance from the patients' point of
view against this giving of presents to nurses.
Now let us look at it from the nurse's point
?of
view. It is taught in all good hospitals that
the friendly 2d. or Id. seed cake must be courteously
refused ; the rule of " no presents " is very strict. It
was one of the things the philanthropist Howard
?complained of so, a hundred years ago, that unless
the patients tipped the nurses they were not properly
-attended to. That is done away with now, and to
the modern nurse the temptation of twopence is not
Very great. But is it any more dignified to accept
1 ls it not just as much a "tip"as fivepence,
the only difference being one not of principle but of
the degree of temptation 1 The accepting of presents
*>y the employed from the employer, especially when
the employment is so temporary as in private nursing,
cannot but be a degradation to any professional
feeling. Cabmen are the only other workers we can
recall who habitually expect, and will accept, more
than their recognised earnings. Domestic servants
will take tips from their masters' friends, and here,
also, you get the feeling of degradation. If wages
"were higher and tips unknown, domestic service
^ould attract worthier men and women.
The trained nurse is always protesting against
being ranked with the domestic servant, and we do not
^hink she holds the cabman as quite her equal \ it is
a pity then that she does not once for all crush out
of her profession one of the worst ills of those other
two callings. It is no use to say that the best
nurses and the best institutions do not allow tips ;
what we want to do is to raise the mass of the
profession to a higher standard, and most cer-
tainly not to countenance what we believe to
be a growing evil. Every hospital and institution
ought to have a definite rule that the nurse is to
accept no present from a patient or a patient's
friends. There are exceptional cases where possibly
a letter of appeal against this may go to the super-
intendent of the institution and be duly considered,
but on the whole the rule ought to be very strict,
and the breaking of it ought to be met on the first
occasion by rebuke, and on the second time by
expulsion. Moreover, nurses working on their own
account ought to make the same definite rule for
themselves ; the nurse's wage now does not need
supplementing in irregular ways, and the honour of
her vocation, which is becoming somewhat shaky in
England, does need supporting in every possible
manner.
Mere words are of no use : those who feel strongly
on this question should at once bring it before the
institution for which they work, and if the rule does
not already exist, suggest that it be inserted. The
subject also is one which could well be brought up at
clubs and societies for debate, and no effort should
be spared to get the whole body of nurses to pull
together in this matter. So long as trained nurses
expect either a gift or an addition to their cheque on
leaving a patient, and let this be known in conversa-
tion, as we fear some of them do, so long will nursing
fail to win the respect of the public. We should
like to know what a free-born independent American
nurse would say, if one of her patients offered her a
present in money or jewellery. She would regard it
as an insult; and are our English nurses in any way
less entitled to justice and respect 1
There is with ourselves much talk of the profession
of nursing, its high calling and the privileges and posi-
tion which the public ought to grant to its members.
What is needed in England to-day is less talk and
more reality and self-respect in practice. The
principles on which alone a great profession can
safely rest its claim to public recognition must in
the first instance strictly govern the actions of all its
members. When trained nurses fully recognise and
act upon this cardinal rule they will obtain for
themselves a position and standing which they can
never otherwise attain to. The best amongst them
must strenuously resist to extermination enervating
practices like " touting for tips " from patients, or
the acceptance of gifts in any form or shape. A fair
remuneration for good services well and faithfully
rendered must ever constitute the bed-rock of safety
for all self-respecting women workers amongst the
sick.
356 Nursing Section. THE HOSPITAL March 28, 1903.
lectures on flDebical anb Surgical nursing.
By John Hopkins, F.R.C.S., Medical Superintendent of the Central London Sick Asjlum District Asylums,
Cleveland Street, W., and Hendon, N.W.
LECTURE IV.?SPECIFIC FEVERS.
This class of diseases is characterised by the readiness
with which they are communicated from one individual to
another. It is supposed that spores of microrganisms are
passed from one to another; that they lie dormant for some
days in the tissues and then burst into rapid growth. The
time during which they fail to make their presence known is
called the period of incubation. Every fever has a different
and distinct period. People who have been exposed to in-
fection are sometimes put into quarantine to prevent them
?conveying it to others, and are kept there under suspicion
until the incubation period has passed by. In the case of
small-pox, all those who have been exposed to it, are vacci-
nated at once, and as vaccination runs a more rapid course
than small-pox, it produces its full effect on the patient
before the small-pox begins to betray its presence, and thus
stops it altogether, or so modifies it as to reduce the danger
from it very greatly.
Small-pox has an incubation period of 12 days. The
onset of the fever is characterised by pain in the back.
With the appearance of the rash, which is on the fourth
day, the temperature falls and rises again when pus forms in
the pocks. There may be only a few? spots, or so many
that they touch; the disease is then said to be confluent.
The rash appears upon the mucous membranes as well as
the skin. Each pock may leave a pitted scar on healing.
In dealing with a case of small-pox the nurse's attentioh
to the rash in the suppurating stage may be of the greatest
value. The fever is largely due to the presence of the pus,
and the more freely this is allowed to discharge, the greater
is the chance of the patient's recovery. Her object
generally must be to prevent the drying up of the discharges
and the formation of scabs. To this end various moist or
oily applications are used, a mask being made for the face,
or warm antiseptic baths are made useiof. Attention to
individual pocks may be sometimes necessary, each of them
being washed out with strong carbolic acid or other
antiseptics.
Chiclien-pox is a mild disease that gives rise to very little
trouble. The spots are scattered about the body, limbs, face
and scalp; look like little blisters on the normal skin and
seldom suppurate or form pus. Sometimes they cause pitting.
In unhealthy children they sometimes lead to sloughing.
The distinction of chicken-pox from small-pox is sometimes
difficult, and whilst doubt remains it is necessary to take all
proper precautions against spread of the infection.
Measles has an incubation period of ten days. The onset
is marked by running at the eyes and nose and by sneezing.
The temperature falls before the rash appears on the fourth
day, and then rises again with the appearance of the rash.
Measles is the most infectious of all the fevers, the sneezing
being a means of scattering the germs of the disease freely
about. Though it is not directly fatal, disease of the
respiratory tract and of the middle ear are common compli-
cations, and it is for these reasons that exposure to a chill is
to be avoided.
Scarlet Ftver has the shortest period of incubation of all
? the fevers. Three days after exposure symptoms may be
expected. The onset is acute, generally accompanied by
vomiting and a rapid rise of temperature. In 24 hours from
the onset the rash appears. The throat is inflamed and
sore, and sometimes gives rise to glandular abscesses or is
invaded by diphtheria. Inflammation of the kidney and
dropsy are to be dreaded. Peeling after scarlet fever is
generally complete in six weeks, the last parts to peel being
the soles of the feet.
In nursing a case of this disease, too much attention
cannot be given to the throat, the object being to destroy
any micro-organisms that find their way there, and are
likely to invade the tissues of the throat. A frequent use
of gargle, spray or syringe is generally required. Carefully
watch for the signs of kidney trouble. (Edema of the
eyelids is the first evidence of the dropsy that results,
which may come on very rapidly, quickly ending in death.
Examination of the urine for the presence of albumen i&
the surest way and the earliest to get an indication of the
state of the kidneys. This should be made every day.
Provided the patient is not exposed to a chill, fresh air is
always desirable, which is best obtained by keeping open a
window. i
Not only is the dissemination of the scales of peeling epi-
dermis prevented to some extent by a daily wash or batb
with some antiseptic solution, and the application of an oily
substance to the skin ; but this is also advantageous to the
patient.
Typhoid fevtr has an incubation period of fourteen days.
Its onset is not marked by any decided symptoms, and the
nature of the case may not be discovered for several days.
The temperature is somewhat characteristic in the early
stage, rising 2? F. at night and falling 1? F. in the morning
until it reaches 103? to 105? F.
The local inflammation in this fever is in the small bowel,
which gives rise to characteristic dejecta, and the bowels
may act very frequently. The inflammation I principally
affects the patches of Peyer'a glands, which slough and per-
foration of the peritoneal covering of the bowel or severe
bajmorrhage may result. When the contents of the bowel
escape into the peritoneal cavity, fatal peritonitis follows.
In nursing typhoid fever special attention is to be given
to the food and to the action of the bowels, and precautions
have to be taken to prevent any undue movement of the
patient.
Milk is the food of a typhoid patient: if congealed in the
stomach it is-liquefied again before it reaches the damaged
part of the bowel. Thus no strain is thrown upon the
muscular coat, and peristaltic action is reduced to a mini-
mum. Beef-tea and pure broth must sometimes be given,
but no solids. From about the fourteenth day of the disease
very special care is needed, as that is the period when
sloughing commences. All unnecessary movements of the
patient are to be avoided. Even the rolling of a patient on
to his side may so disturb the relative position of the coils
of intestine as to cause perforation. Haemorrhage from the
bowel may be profuse and fatal. A sudden fall of tempera-
ture is never a good indication in this as it is in some other
diseases. It never means a critical recovery, and it generally
signifies severe haemorrhage into the lumen of the bowel.
Bed-sores must be specially guarded against, as convales-
cence is protracted, and a relapse may occur, when the
whole course of the disease is run through again. Hyper-
pyrexia is to be treated by sponging baths, etc.
The clothing should be light and the air of the room cool,
for it is not good that the patient's body should be made to
retain too much of the heat it is so rapidly producing.
Fevers are rendered the more dangerous by the many com-
plications they are liable to. The body of a fever patient is
favourable soil for the growth of micro-organisms, to which
March 28, 1903. THE HOSPITAL.  Nursing Section. 357
axe attributable most of the inflammations that are experi-
enced in fevers. Diphtheria, lymphodenitis, erysipelas,
abscesses, boils, gangrene, rheumatic fever, endocarditis,
pericarditis, bronchitis, pneumonia, pleurisy, nephritis,
diseases of the skin and of the eyes and ears, paralysis, de-
lirium, and mania are some of these troubles. Bedsores
once formed afford a ready inlet for microbic invasions.
Hence the nurse that intelligently combats against them will
be rewarded where others would fail.
Not the least important function is the prevention of in-
fection spreading to others. What comes from the patient
may, and often does, carry the germs of the disease.
Whether his breath is ever infectious is very doubtful, but
any act of coughing or sneezing sprays the air with liquid
particles that may be carried in it a great distance,
conveying the infection with them. Therefore it is necessary
to pay special attention to cases troubled with a cough or a
catarrh that excites sneezing. The exfoliations of the skin,
the dejecta, the saliva that adheres to cup or spoon, or that
may be adherent to his body or bed clothing, or anything
that he handles, may convey infection. Hence the furnish-
ing and the attention required for the sick-room of one
suffering from an infectious disease calls for particular
knowledge on the part of the nurse.
a Xeague of fffiierc? flDeeting at Ikenstngton.
IMPRESSIONS OF A NURSE.
The announcement that under the auspices of the League
of Mercy Sir Henry Burdett would deliver an address on
" Life in the Hospitals," and illustrate it by a series of living
pictures, at Kensington Town Hall, on Thursday night last
week, afforded me the opportunity I had been waiting for
ever since I had heard of the meetings which have been
such a feature of the winter months.
The Audience.
The hour fixed for the Duke of Argyll to take the chair
was half-past eight, and arriving, as I thought, sufficiently
early at a quarter past, I was rather dismayed to find nearly
all the seats in the large room occupied. There were, how-
ever, many nurses who came later than myself, but their
uniform, perhaps suggesting that their time was not at
their own disposal, the officials?who seemed indefatigable
in looking after everybody?provided them .with places, two
or three being invited, as a last resort, to take seats at the
table supposed to be sacred to the representatives of the
press. When the Duke of Argyll, President of the South
Kensington District, mounted the platform and introduced
Sir Henry, the hall was so densely packed that the gangways
at the side and the back of the gallery, were filled with
people standing. Afterwards I heard that hundreds were
turned away from the doors. Looking round before the lights
were switched off, I could not help thinking that, though
some of the few reserved benches contained Dora, Countess
of Chesterfield, Lady President of the South Kensington
district, and her friends, the rest of the space being free to
all, the bulk of the audience consisted of the very class
whom the speaker most wanted to reach, namely, those who,
save in exceptional cases, would not apply for admission to
the hospitals themselves, and are yet unable to help the
charities by large donations, though probably in a position
to render personal service. The Chairman seemed to be of
the same opinion, for in his brief but emphatic speech, he
dwelt upon the importance of personal service as well as
service of the purse.
A Universal Duty.
For the benefit of those who did not know why he should
come forward on behalf of the hospitals, Sir Henry said that
all his life had been spent among them, and that he had lived
in- them for nearly fifteen years, though it was not until he
frad a serious illness himself and was nursed in the insti-
tution that he realised something of the aggregate of all the
suffering which surrounded him within those four walls. He
then went on to show how the rich patient, attended by the
dost famous physician, nursed in luxury at home by the most
skilful nurses, owed as big a debt of gratitude to the hos-
pitals as the poor in the wards. " It does not matter, he
?aid, " how rich a man may be, how poor a man may be,
tieh and poor alike are dependent upon the hospitals. Every
man and woman has a duty in days of health to render to
the sick, and it is a privilege to be able to fulfil it."
The Out-patients.
Having thus taken his audience into his confidence, and
afforded them a clue to the object in view, the lecturer gave
the signal for the lights to be switched off, and the first
picture |was thrown upon the sheet. This was a representa-
tion of a London slum, from whose cellars and garrets many
of the hospital patients are drawn. In striking contrast,
the second and third pictures were St. Thomas's Hospital
from two different points of view; then came " Big Ben "
and the Terrace on the opposite side of the river. Next fol-
lowed the entrance to " Bart's," and Sir Henry had a few
words to say about the scheme for rebuilding. As I rather
expected, the entrance to Guy's succeeded, and then came
the familiar interior of an out-patients' department. The
particular hospital in this instance was the Seamen's, at
Greenwich, with the patients representing many nationalities
and diverse colours. Here an amusing anecdote was related.
A patient, the lecturer said, came to the hospital one day, and
though the staff prided themselves upon beiDg good linguists,
no one could understand him. However, the doctor overhauled
him, ordered him to have a good bath, had his hair cut, and
sent him to bed. The next day it was discovered that he
was only a visitor who had come to see an inmate! In rapid
succession were pictures of the women's out-patient depart-
ment, the refreshment bar, the examination of a patient
by the doctor surrounded by students, the dental depart-
ment at " Guy's," the final picture in this set showing the
patient going to the dispensary to get the medicine.
Life in the Wards. .
The second set of pictures dealt with the in-patients,
starting with a street accident, and at this stage the lecturer
put in a strong plea for emergency beds in which patients
can be placed until it can be ascertained whether they are
" drunk or dying." Next a railway accident was portrayed
on the sheet, and then one of the victims was seen through
the various stages of transfer until he was comfortably
settled in bed. " Life in the Wards," the third set,
included a hospital ward a hundred years ago, a horribly
cheerless sort of barrack ; one of to-day with every modern
improvement?nurses making beds, the house surgeon" look-
ing in upon a tracheotomy case, and the visiting physician
going his rounds. I could not help laughing when Sir
Henry pointed out to the audience which of the two female
figures was nurse and which was sister. The latter, he
remarked, would be easily recognised because she carried the
badge of office, the inkpot, adding, that " if, except under
her auspices, a spot got on- to the ? floor, next day someone
would die." ? - ? - - ' :
358 Nursing Section. THE HOSPITAL. Makch 28, 1903.
A LEAGUE OF MERCY MEETING?Continued.
Food and Physic.
There were two pictures of dinner time, one showing a
convalescent sitting at table, and the other a patient in bed
on fall diet. After "food" came "physic," and after
"physic" came "temperature," in all of which the nurse
was prominent. In this set were also several pictures of
children, which appeared to excite particular interest,
especially " the pet of the ward," and the lecturer expressed
his approval of the presence of a few children in a women's
ward, though he did not think it desirable that they should
be included in a male ward. Other pictures which strongly
appealed to me were the singing of the evening hymn in
the male ward, and one in which Queen Alexandra and her
daughters were delighting the patients at Brompton Con-
sumption Hospital with a musical entertainment. This,
perhaps, more than any other brought down "the house,"
the Court suburb being naturally enthusiastically loyal.
The Modern Operation Theatre.
" Visiting Day " showed the friends without and within the
hospital gates, and the next set, paiofully but necessarily
realistic, illustrated the horrors of surgery before the days
of chloroform, and led up to a modern operation theatre in
the Johns Hopkins Hospital at Baltimore, which was in turn
succeeded by a very remarkable picture of a patient who
was under anaesthetics for two and a-half hours in a
great provincial infirmary, with six doctors and three or
four nurses in attendance. Wonderful, too, to state, the
case was quite successful. The Convalescent Home was a
pleasant variation, and I learnt for the first time that Mr.
Peter Eeed provided and furnished at Swanley, in a beautiful
position on the Kentish hills, an institution for surgical cases
likely to derive benefit from plenty of fresh air.
Mrs. Gamp and the Nurse of To-day.
The feature of the "Sisters and Nurses" was a pair of
pictures, one representing Mrs. Gamp, and the other a
modern nurse, which the lecturer, who throughout spoke
very kindly of the modern nurse, said were emblematical of
the progress made in all departments of hospital life
between 1870 and 1903. Of course the series would not
have been complete without a representation of a ward
decorated for Christmas; and finally the lecturer took his
audience to South Africa, and thowed them the exterior
and interior of a hospital on the veld, expressing, amid
loud applause, his conviction that there never was a war in
which the sick and wounded received so much skilled treat-
ment and tender care from the doctors and nurses.
A Splendid Precedent.
The practical end in view was to persuade everyone
present to help the hospitals by joining the League of
Mercy, either as vice-presidents, members, or associates ; and
Sir Henry having explained how this might be done, asked
us all to individually repeat the act of the Good Samaritan.
Judging by the forest of hands enthusiastically held up in
response to the suggestion that those who were willing to
assist should give this sign, the effect produced by the
lecture must have been most promising for the future of
the League of Mercy in Kensington.
ZIbe 36r(tisl5 ?^narcological Society.
NURSING EXAMINATIONS.
The conditions of the nursing examinations by the
British Gynaecological Society are as follow:?
1. Until December 31st, 1903, candidates for examinations
will be required to produce (?) a certificate of not less than
one year's training, satisfactory to the Board of Examiners,
from a general hospital containing not less than 40 beds;
(&) a certificate of training in a lying-in institution, or in a
hospital for women, or in the gynaecological wards of an
institution approved by the Board of Examiners; (<?) three
recent testimonials of character.
2. During the year 1904, candidates will be required to
produce (a) a certificate of two years' training in a general
hospital containing not less than 40 beds ; (J) a certificate
of at least three months' training in a lying-in institution, or
in a hospital for women, or in the gynaecological wards of an
institution approved by the Board of Examiners; (c) three
recent testimonials of character.
3. After December 31st, 1904, all candidates will be
required to produce (?) a certificate granted, after three
years' training, by a general hospital containing not less than
40 beds; (b) a certificate of at least three months' training
in a lying-in institution, or in a hospital for women, or in the
gynecological wards of an institution approved by the
Board of Examiners; (c) three recent testimonials of
character.
Subjects of Examination.
1. The examination will be divided into two parts?(a) a
written paper; and (J) vivd voce.
2. During the year 1903 the written paper will consist of
eight questions upon medical and theoretical details, (a) For
the Maternity Nursing Examination these will comprise a
practical knowledge of antiseptic principles and of the
sterilisation of appliances; the preparation of the mother
and of her room before labour; the duties of the nurse
during labour; the duties of the nurse after labour in the
case of mother and child, including the preparation of diet&
for each; the precautions and duties of the first fortnight
and of the second fortnight; the accidents, diseases, and
complications met with before, during, and after labour, and
the nurse's duties in each event; the writing out of reports,
of a maternity case. (J) For the Gynaecological Nursing-
Examination?A practical knowledge of antiseptic prin-
ciples and of the sterilisation of appliances; a knowledge
of the more common diseases of women which require
nursing; a knowledge of the methods of preparing diets
for operation and other cases ; the methods of preparing a*
patient for a vaginal, and for an abdominal, operation ; the
duties of the nurse during and after such operations; a
practical knowledge of the subsequent progress of such
patients, and of the duty of the nurse in various emer-
gencies; the writing out of reports of a gynaecological
case.
3. The vied voce examination will be conducted in two-
parts.
Part I.?Practical nursing detail, such as the making of
beds for different cases; the proper lifting or moving of the
patient; the proper washing and dressing of the infant
the administration of medicines, enemata, douches, etc.
This part will be conducted by the hospital matrons who are
examiners.
Part II.?Theoretical nursing: The names and use of
ordinary instruments ; the preparation of antiseptic lotions;
and dressings; the methods of preparing plugs, bandages,
and dressings; the proper charting of temperature, pulse,
etc.; questions elucidating the candidate's answers to the
written papers. This part will be conducted by the medical
examiners.
Fees.
The fee payable by each candidate either for the Monthly
Nursing Certificate or for the Gynaecological Nursing Certifi-
cate will be one guinea, of which 7s. will be returnable to
her if she fails to pass the examination. The examinations-
in 1903 will be held in the first week of June, of September,,
and of December. The names of candidates for the
examination in June should be sent as soon as possible?and
all further information can be obtained by sending a stamped
and addressed envelope to Dr. Aarons, 14 Stratford Place,.
London, W, -
March 28, 1903. THE HOSPITAL. Nursing Section. 359
Uaby Xonfionberr? anb district
IRursing.
The friends and supporters of the Shoreditch and Bethnal
?Green District Nursing Association assembled in such goodly
numbers at the annual meeting last week that the stately
apartment at Londonderry House, where by the kind invita-
tion of the Marchioness of Londonderry it was held, was
completely filled.
Lady Londonderry herself took the chair, and a letter was
read from Princess Christian, the President of the Associa-
tion, stating how much she regretted that she was unable to
attend the meeting.
Mr. James Langton, F.R.C.S., in moving the adoption of the
report, said that associations of this kind were particularly
welcome in large cities such as London, and especially in
poor neighbourhoods. He proceeded to trace the history of
nursing from its inception at the end of the fourth century
up to the present day, and alluded to the splendid work per-
formed by Miss Florence Nightingale during the Crimean
War. England was proud of her nurses, and well she might
be, for they never failed to come forward in the hoar of
danger and perplexity. The country owed them a great
debt for raising the moral standard of the sick poor whom
they visited.
Mrs. James Stuart gave some touching instances from
personal knowledge of cases among the very poor where the
district nurse had come to the rescue and rendered most
valuable assistance. The distiict nurses, besides all their
other qualifications, must possess tact. The superintendent
of their association always made a point of impressing upon
the new nurses how important it was to take great, care of
the little belongings of the poor.
Lady Londonderry, in acknowledging a vote of thanks
accorded to her, said it gave her great pleasure to be able
to assist the Association in the smallest possible way.
District nursing, she continued, was started quite twenty
years ago, but the belief that every woman was a natural
nurse died very hard. She was herself connected with a
great many district associations in all parts of the country,
and considered that the benefits conferred by them were in-
calculable. In four special directions the nurses rendered
immense services to the poor. The first was in keeping the
home together ; for instance, when the mother of a family fell
ill she greatly resented the idea of being obliged to leave
home. The second great service rendered by the nurses was
hy the reduction of infantile mortality, starting the children
fairly in the world and caring properly for them during the
first month of life. The third was the prevention of serious
illness by taking the proverbial stitch in time; and the fourth
?service was that the nurses took with them an atmo-
sphere of refinement into the poor homes they visited. Lady
?Londonderry appealed to all present to help the work, and
'then went on to recommend to the Shoreditch Association
"the plan which was adopted in many great towns where
there were large factories of obtaining the co-operation of
the workmen. This was done by stopping with their consent
a penny per fortnight from their wages, and when possible,
a*ter they had saved a certain sum, they were allowed to
olect a representative on to the committee. This plan had
been found to be productive of much benefit. Lady
Londonderry pointed out, in conclusion, that the committee
the Association state in their report that in spite of much
kind help in the past, largely increased subscriptions were
deeded if the work was to be carried on satisfactorily, and
an additional ?100 a year in subscriptions was urgently
required.
Opening of tbe IRcw Mart) at (Su^'s
Ibospital.
A very pleasant function of a semi-private character took
place at Guy's Hospital last Friday, when Mrs. Walter
Farquhar opened the renovated and now beautiful ward
known as Job, in memory of her late husband. The work of
restoration begun some time back, and had ceased for lack
of funds. It is owing to the generosity of Mrs. Farquhar
and her son that the ward is now completed and occupied
by male surgical patients. Like all the surgical wards, it is
in the shape of the letter |_ > the colouring?the choice of
the sister?is cream, with a dado of soft green, and the
scheme is continued in the kitchen and bath-room, which
open out of the ward, with this difference, that the walls
above the dado are of white tiles. The sister's bedroom and
sitting-room, also opening out of the ward, but at the
opposite end, have the same colouring, and are very cosy
little apartments. There is plenty of cupboard room at this
end of the ward, where numbers of small brass handles
denote that each nurse has her own locker for ward purposes,
and there is ample room for the storage of ward linen.
A patients' tea had been arranged to celebrate the com-
pletion of the alterations, and as the tables were daintily
decorated with spring flowers?narcissi, daffodils, mimosa,
and Neapolitan violets?the scene in the afternoon sunshine,
was very bright. The matron, Miss S. A. Swift, and several
of the sisters and nurses from other wards were present, as
well as some of the medical students and a few specially
invited guests.
Dr. C. J. Symonds, in a little address of welcome to the
donor, drew special attention to the floor, which is of wooden
parquetry. It was an example, he said, of what a ward floor
should be, and all concerned, whether doctors or nurses,
could assure Mrs. Farquhar that she had given them a
delightful place in which to work. He thanked her on
behalf of the sister and all the nurses. The treasurer, Mr.
H. Cosmo Bonsor, said that Dr. Sjmonds had alluded to
the floor; he wished to draw attention to the ceiling, which
was absolutely fireproof. It was of uralite, a mixture of
asbestos and chalk, and came from Russia in blocks; these
had been painted white, and made a ceiling well adapted for
a modern scientific ward. Mr. Bonsor concluded with a refer-
ence to the repoussSe tablet let into the wall, which com-
memorates the completion of the alterations, in memory of
Mr. Walter Farquhar.
Mrs. Farquhar declared the ward open in her own and her
son's name, and then tea was dispensed by the sister, assisted
by the students and nurses.
The reappropriation of wards which is rendered possible
by the completion of Job appears somewhat complicated
to the uninitiated; it consists in the exchanging of male and
female medical wards, by which a new female ward is made
available; while a number of beds long closed for lack of
funds have been reopened, owing to the large grant (?5,000
annually) made by King Edward's Hospital Fund for the
purpose. There is a total gain of 50 beds.
The two female wards will be above the ophthalmic
wards, and the male wards over the out-patients depart-
ment. The following appointments have been made: Esther
Ward, sister, Miss Trail (at present Sister Mary) ; Stephen
Ward, sister, Miss Jones (the present sister); Philip Ward,
Miss Godby (the present sister). The appointment of
sister to the new Mary Ward has not yet been made. As
soon as the walls have been colour-washed, these wards will
be put into occupation, and the sisters and nurses will enter
upon their duties. Owing to a re-arrangement of the medical
and surgical staff, each ward will now be visited by two
senior or two junior physicians or surgeons only.
360 Nursing Section. THE HOSPITAL. March 28, 1903.
jEverp6o&?'s ?pinion.
BURY UNION HOSPITAL, LANCASHIRE.
" Miss Esther A. Thompson," the new staff nurse at Bury
Union Hospital, writes : In your issue of March 7th you state
that I was trained at Prestwich Union Hospital. I was
trained for three years at St. Olave's Infirmary, Rotherhithe,
where I also held the post of charge nurse for eleven months.
I have since been staff nurse at the Prestwich Union
Hospital.
[The appointment was inserted in accordance with the
particulars sent to us by the authorities at Bury.?Ed.
The Hospital]
GLASGOW AND WEST OF SCOTLAND "CO-OPERA-
TION OF TRAINED NURSES."
" One of the Staff " writes : As one of the nursing staff
of the " Glasgow Co-operatidn," therefore a member of that
association, I beg leave to inform you and readers of The
Hospital that the statements that have appeared lately
?regarding this institution are misleading and incorrect.
Twice, we have figured in your magazine as a "sham co-
operation," but how can the word sham be applied to an
association whose workings are all open and above board,
and whose members are all duly informed of the same? I
have been a member for nearly four years, and although
seldom able to be present at the meetings, have always been
previously informed when and, where those meetings were to
be, and I am no privileged member. Then what does our
friend (so-called) Mr. Bannatyne mean when he calls us
servants? True, all members of the co-operation are
servants?servants of the co-operation and servants one
of another, and none serve the association more, I should
say, than those members who are on the Executive Committee.
It is to their labours and the deep interest they have taken
in the welfare of the nurses that we owe the success of our
co-operation. Others have tried to raise and maintain a
nursing institution on the co-operative system, without a
committee, and what has always been the tesult? They
have failed in their expectations They are not successful.
And why 1 Because the nurses have tried to do for them-
selves what our executive committee has done for us. How
can nurses, whose duty it is to nurse the sick, attend to
business ? Scattered all over the kingdom as well as on the
Continent it is well for us to know that our affairs are being
attended to the while, and that, too, by a body of men and
women in whom we have every confidence. Knowing what
they have done for us in the past we do not fear to trust them
with our future. A great deal has been f aid about our new con-
stitution, about its being forced upon us without our sanction,
and how that under its dispensation we are liable to be dis-
missed at any moment. The first statement is untrue ; the
second is utter nonsense. Some weeks before the general
meeting in November a draft of the new constitution was
placed in the office and in the nurses' parlour, and their
attention drawn to it by the lady superintendent; further-
more, a notice was put in the Glasgow Herald intimating
that copies were to be had at the office, 18 Sardinia Terrace,
and many of the nurses availed themselves of this oppor-
tunity of reading and studying its contents, and general
approval was the result. However, at the general meeting a
number of nurses were present who had not seen the draft
of the new constitution, or who, having seen it, had not
taken much notice of its contents, so when at this meeting
Mr. Bannatyne moved a motion condemning it, accom-
panied by a great deal of flowery language that the nurses
were being done out of their rights, etc., etc., because they
had not been given an opportunity of perusing this docu-
ment, the majority of nurses present, thinking that a
member of the executive committee who differed from other
members was speaking on their behalf, voted for Mr.
Bannatyne's motion. Accordingly, although the executive
committee were well aware that the nurses were being
misled, the meeting was adjourned, whilst it was decided
that each nurse should be furnished with a copy of the new
constitution. This was done. A copy was sent to each
nurse along with a letter asking her to read care-
fully the various clauses, and soliciting her approval or
disapproval of the same with ithe result?so far as I am
aware, and I have met a great many of the nurses since
then?that it met with their unanimous approval. Referring
to that other remark, that we are liable to be dismissed on
shortest notice, the following is the standing rule" Should
a serious charge be brought against any nurse, she shall be
suspended until next meeting of executive committee, and
no nurse shall be dismissed without first being given an
opportunity of defending herself." Now what does Mr.
Bannatyne want 1 That each nurse be a law unto herself,
breeding disorder and confusion, and finally dissolution 1
He is no friend of our association; rather, I think he is, or
tried to be, our enemy, and I fear personal feeling has had
more to do with his zeal on our behalf than friendship. He
is not a member of the Co-operation ; doubtless he became
possessed of a member's ticket, the right of admission to our
general meeting, but he had no right to use that ticket,
Personally, I think it a pity that he was not admitted,
because I am positive that his efforts to prevent the passing
of the new constitution a second time would have been
futile.
appointments,
[No charge is made for announcements under this nead,and we aie
always glad to receive, and publish, appointments. But it is
essential that in all cases the school of training should be
given.]
British Lying-in Hospital, Endell Street, W.C.?
Mrs. Low has been appointed second assistant matron. She
was trained at the British Lying-in Hospital, in midwifery
and monthly nursing, in 1901, and has since been occupied
in private nursing. She will be responsible for the out-
patients' department, which includes visits to patients'
homes, in charge of the pupils
Fever Hospital, Stockton-on-Tees.?Miss Lydia H.
Ritson has been appointed charge nurse. She was trained
at Chesterfield and North Derbyshire Hospital, and has since
been staff nurse at Sheffield Royal Infirmary and Kendal
Hospital; also sister of the theatre and male surgical ward
at Hartlepool Hospital.
Hastings Union.?Miss Louisa Soley has been appointed
assistant nurse. She was trained by the Meath Workhouse
Nursing Association at the Mildmay Memorial Hospital,
and has served as assistant nurse at the Totnes Union
Infirmary.
Hertford and Ware Joint Hospital.?Miss H. E.
Cock has been appointed matron. She was trained at the
City Hospital, Birmingham, and has since been matron at
the Isolation Hospital, Cheshunt.
Mount Vernon Consumption Hospital, Hampstead.?
Miss Grace Edith Jarrett has been appointed staff nurse.
She was trained at Addenbrooke's Hospital, Cambridge, and
has since done private nursing on the staff of the West-
minster Home.
Sheffield Royal Hospital.? Miss A. L. Earle has
been appointed lady superintendent. She was trained at
Liverpool Royal Infirmary, and has since been in succession
sister, night superintendent, and assistant lady superin-
tendent in the same institution. She has also had experi-
ence on the private nursing staff of the Liverpool Infirmary
for two years.
Western General Dispensary, Maryleeone Road,
N.W Miss Rose Lena Shappere has been appointed matron.
She was trained at the Prince Alfred Hospital, Melbourne,
Victoria, and has since been sister at the Perth Hospital and
Kalgoorlie Mine Hospital, Western Australia, and night
sister at the Adelaide Hospital, South Australia. She has
done private nursing for some jeais in New Zealand and
Melbourne. She also nursed the sick and wounded during
part of the war in South Africa and went through the siege
of Ladysmith.
March 28, 1903. THE HOSPITAL. Nursing Section. 361
Echoes from tbc ?utstbe mnorlfc.
Movements of Royalty.
Ox Monday the King held an Investiture of the Imperial
Service Order, the Prince of Wales being present in the
Throne Room at Buckingham Palace with his Majesty.
The decoration, it will be remembered, was recently created,
and is intended mainly as a reward for distinction in any of
the numerous branches of the Civil Service. The recipients
of the Order included the Prince of Wales and one lady,
Miss Constance Smith, of the Post Office Savings Bank.
The afternoon of March 21st, 1903, is not likely to be
soon forgotten by the elder children of the Prince and
Princess of Wales. For the first time in their lives Prince
Edward, Prince Albert, and Princess Mary were allowed to
go to the theatre, and were taken by their aunt and uncle,
Prince and Princess Charles of Denmark. " Mother Goose "
at Drury Lane was the piece selected, and the Royal party
arrived just after the curtain had risen. The three children
occupied the front chairs in the box, Prince and Princess
Charles being seated behind. Apparently all the marvellous
sights required frequent explanations, for continual ques-
tions were asked, some of which, by the laughing rejoinders,
must have been of an amusing character. The little boys
took their pleasure less enthusiastically than their small
sister, who evidently enjoyed applauding almost as much
as witnessing the dancing and the acting. Between the
parts tea was provided by the manager in the ante-room,
and bread-and-butter formed the substantial foundation of
the meal. The antics of the clown and pantaloon later on
elicited much laughter, and when the former threw four
extra large crackers into the Royal box the glee of the
youthful sightseers was unmistakable. Before leaving the
theatre the two little Princes and their sister were presented
with an album containing photographs of the principal
performers.
Reception to Mr. Chamberlain.
On Friday last Mr. Chamberlain was presented by the
City Corporation with an address of congratulation on his
return from his mission to South Africa. A large and dis-
tinguished company assembled at the Guildhall, and after
the presentation had been made by the Lord Mayor, the
Colonial Secretary, in reply, fresh from his experiences,
repeated the assurances he gave when the war was in
Progress, that when it ended it would bring an enduring
peace and a well-justified hope of a strong, united, and
Prosperous South Africa. He declared his belief that the
Transvaal and the Orange River Colony would be among the
r^ost prosperous dominions of the Crown, and after warmly
eulogising the services of Lord Milner, he urged that our
policy must be a continuous one?not that of a party, but of
the whole nation. At the close of the proceedings Mr.
Chamberlain, accompanied by his wife, drove in the Lord
Mayor's carriage to the Mansion House, where they were
entertained at luncheon. In response to the toast of his
ealth the Colonial Secretary said that he had felt for some
time that this was a critical period in our history and would
Probably settle for ever the question whether the Empire
^as '? be consolidated and maintained, or whether it was to
roP apart into separate atoms, each caring only for its local
^d parochial concerns. Mr. Balfour subsequently affirmed
at Mr. Chamberlain's mission "had made the relationship
etween the Mother Country and the colonies a flesh-and-
?d communication." Mr. Chamberlain has refused a
great public welcome at Olympia.
Death of Dean Farrar.
?N Sunday evening Dr. Farrar, Dean of Canterbury, died
* the Deanery, Canterbury. He had been ill for some time
r?m creeping paralysis, but the end cam* rather unex-
/
pectedly. On Saturday morning the Dean was present at
the Cathedral service, and in the afternoon he drove with
Mrs. Farrar to the St. Lawrence Cricket Ground, where the
King's School sports were in progress. He had a restless
night, and got worse during Sunday, passing away peace-
fully at seven o'clock. The^ prayers of the congregation
were asked for the bereaved family at the Cathedral service.
Dean Farrar was well known as a popular preacher and author.
He was in succession master of Marlborough College, Canon
of Westminster Abbey, and Archdeacon of Westminster, before
his appointment, in 1895, to Canterbury. His most cele-
brated books were his " Life of Christ," " The Early Days of
Christianity," the "Life of St. Paul," and "Eric; or.
Little by Little," a tale of schoolboy life which was pub-
lished as far back as 1858.
Cremation and Crime.
Me. Justice Grantham, in summing up the evidence in
the Southwark poisoning case, which resulted in a verdict of
wilful murder against the man known as George Chapman,
said.- " Were it not, fortunately, tha1; cremation had not been
adopted in the case of Chapman's victims, there would have
been no possibility of the cause of death being elicited";
and he added that, " if bodies were burned, people might
depend upon it, that the practice would tend to increase con-
siderably the number of deaths from poisoning." To these
remarks, the president of the Cremation Society, Sir Henry
Thompson, has replied that "it is a fact well known to
medical men who have been conversant with the subject,
that any poisoner who may attempt to get rid of his victim
by sending him to a crematorium in this country, is almost
certain to be detected." This fact, he contends, " was
admitted and appreciated by the committee of the House of
Commons which Mr. Asquith early in April, 1893, granted on
the subject after it had been brought before him by a deputa-
tion ; and the evidence on all sides of the question, as well
as the details of the system of minute inquiries made and
adopted in every case, has been embodied in a Blue-book
which was printed the same year."
A German Play at the St. James's Theatre.
" Old Heidelberg," which was produced, last week at
the St. James's Theatre, is an English version of a popular
modern German play bearing a similar title. It is a simple
story of love and duty, but it is told in a sympathetio
manner, and the style is fresh and breezy. Five acts
are needed to show how the young Hereditary Prince of
Sachsen-Karlsburg, under the care of his elderly tutor, Dr.
Jiittner, comes to Heidelberg to join the students for a year,
and so learns not only the book lore which he might possibly
have acquired at the stiff slow Court, but a knowledge of
human nature of which he had hitherto been quite ignorant.
The niece of the landlord of the inn at which the mirth-
loving students hold high revel is very pretty and very
winsome, and for four months the Prince and the girl see
each other frequently. Then comes a summons back to
Karlsburg to take up the reins of government. A couple of
years later, a satisfactory matrimonial arrangement having
been made for the Prince, he has a great desire to see the
place where he spent the happiest months of his life. His
wish is realised, but he finds that most things have changed
and have lost their brightness except little Kathie, who makes
no secret of her love for him. But duty calls another way,
and the scion of a noble house returns to his court to wed
the lady of his own rank, whilst Kathie determines to make
the cab proprietor in Vienna as happy as may be. Mr.
Alexander is delightful as the Prince, and Miss Eva Moore
perfectly charming as the Maid of the Inn. Mr. J. D.
Beveridge is a lovable old tutor, and Mr. E. Lyall Swete a
capital supercilious valet.
362 Nursing Section. THE HOSPITAL. March 28, 1903.
for IReatung to tbe Slcft.
" LOOKING UNTO JESUS."
TO Thee, 0 Jesu, I direct my eyes,
To Thee my hands, to Thee my humble knees,
To Thee my heart shall offer sacrifice,
To Thee my thoughts, Who my thoughts only sees:
To Thee myself, myself and all I give,
To Thee I die, to Thee I only live.
Sir IF. Raleigh.
Give me not what I ask, but what is good,
Merciful Saviour, unto Thee I look,
Oh, teach me these repining thoughts to brook.
. . . Make me Thine own
And take me: of myself I am afraid,
Oh,-take me from myself; oh, take away
Whate er of self is in me, and, I pray,
Give me on what my spirit may be stay'd,
And that I know full well is but Thyself alone.
T. Williams.
Grant to us, Lord, in all our difficulties, Thy help ; in all
our perplexities, Thy counsel; in ,all our dangers, Thy pro-
tection ; in all our sorrows, Thy peace. Amen.
Anon.
Obedience must often be blind?obedience in the dark
not only when we see the whole path marked out before us,
but when we can discern nothing beyond the next step we
have to take. When God is leading His children to victory,
He has His own way of securing it; and the steps towards it
often look more like defeat than triumph. A. P.
He has an especial tenderness of love towards thee for
that thou art in the dark and hast no light, and His heart is
glad when thou dost arise and say, " I will go to my Father."
For He sees thee through all the gloom through which thou
canst not see Him. Say to Him, " My God, I am very dull
and low and hard; but Thou art wise and high and tender,
and Thou art my God. I am Thy child. Forsake me not."
Then fold the arms of thy faith, and wait in quietness until
light goes up in the darkness. Macdonald.
Be still, sad soul! lift thou no passionate cry,
But spread the desert of thy being bare
To the full searching of the All-seeing Eye
Wait?and through dark misgiving, black despair,
God will come down in pity, and fill the dry
Dead place with light and life and vernal air.
J. C. Shairp.
Without holiness "no man shall see the Lord." Is my
first thought how I may please God 1 The faintest desires
are accepted and blessed by Him, much more the steadfast
purpose, the great longing to be what He would have us.
His yoke is easy and His burden light, because He lays the
yoke upon us, fitting it to our strength exactly, and because
He Himself bears our burdens and walks always by our side.
R. TP. Randall.
IRotes anb (Suedes.
The Editor is always willing to answer la this column, with oat
any fee, all reasonable questions, as soon as possible.
But the following rules must be carefully observed
I. Every communication must be accompanied by the Bam
and address of the writer.
s. The question mnst always bear upon nursing, directly It
indirectly.
If an answer is required by letter a fee of half-a-crown must ba
enclosed with the note containing the inquiry, and we cannot
undertake to forward letters addressed to correspondents making
Inquiries. It is therefore requested that our readers will not
?ndoae either a stamp or a stamped envelope.
Alexandria.
(185) Can you give me any information concerning the Victoria
House, Alexandria, and also tell me if there are any English
nursing homes in Alexandria, Egypt ??Nurse M.
Perhaps the English Chaplain at Alexandria could assist you.
French and Flemish.
(18G) I should be glad of the names of books on nursing
published in French and in Flemish ??Matron.
Write to Messrs. Hachette and Co., foreign publishers, Charing
Cross, W.C.
Rubber.
(187) D. M. would like to know whether there is any way of
preserving the rubber of water beds, air pillows, etc. Would
inunction prevent its cracking and perishing ?
Write to Messrs. Bailey and Co., 38 Oxford Street, W., who will
give you instructions.
King's Home.
(188) Will you kindly inform me through your paper of the
address of the convalescent home or homes given by the King for
the benefit of wounded officers from South Africa ??J. C. IF.
Osborne is the only convalescent home given by the King for
the benefit of wounded officers.
Address.
(189) Will you kindly give me the address of the Midwives'
Board, as I should like to apply for one of the vacancies by return
of post ? I am district midwife for , employed by the County
Council, and shouldlike to say somethingon the subject.?Nurse M-
Possibly the Midwives' Institute, 12 Buckingham Street, Strand,
W.C., might help you.
South Africa.
(190) Will you kindly tell me the names of nursing homes in
South Africa ??Morniel.
Apply the Victoria .Nurses' Institute, Capetown, Cape Colony,
and the Lady Superintendent, the Co-operative Residential Club,
Johannesburg.
Convalescent Work.
(191) Please tell me what steps to take in order to obtain
work as a nurse in a convalescent home ? Could I get an
appointment through this paper, or is there another which has
more advertisements respecting convalescent homes ??M. M. G.
Advertisements in this paper constitute the best method of
securing attention to your requirements. You should watch our
columns.
Baby Carrier.
(192) 1. Can you tell me where the broad bands, or slings, which
are used by some children's nurses when carrying babies out of
door, can be obtained ? 2. Also can you tell'me of any general
or lying-in hospital where a trained nurse could receive a thorough
post-graduate course ??A. H.
1. You would be able to obtain a baby-carrier at Whitelev's>
Westbourne Grove, W., and also at S. T. Matthews and Co??
9Ga Vittoria Street, Birmingham, price 2s. Cd. 2. Write to the
Midwives' Institute, 12 Buckingham Street, Strand, W.C.
Standard Nursing Manuals.
"The Nursing Profession : IIow and Where to Train." 2s. net;
2s. 4d. post free.
"Nursing : Its Theory and Practice." (Revised Edition). 3*.6d.
post free.
" Surgical Ward Work and Nursing." (Revised Edition). 3s. 6d?
net.; 3s. 10s. post free.
" Practical Handbook of Midwifery." (New Edition). 6s- net'
6s. 3d. post free.
"Notes on Pharmacy and Dispensing for Nurses." Is. post fr?e'
" Fevers and Infectious Diseases." Is. post free.
" The Art of Massage." (New Edition). 6s. post free.

				

